# A Systematic Review of MicroRNAs in Hemorrhagic Neurovascular Disease: Cerebral Cavernous Malformations as a Paradigm

**DOI:** 10.3390/ijms26083794

**Published:** 2025-04-17

**Authors:** Roberto J. Alcazar-Felix, Aditya Jhaveri, Javed Iqbal, Abhinav Srinath, Carolyn Bennett, Akash Bindal, Diana Vera Cruz, Sharbel Romanos, Stephanie Hage, Agnieszka Stadnik, Justine Lee, Rhonda Lightle, Robert Shenkar, Janne Koskimäki, Sean P. Polster, Romuald Girard, Issam A. Awad

**Affiliations:** 1 Neurovascular Surgery Program, Department of Neurological Surgery, University of Chicago Medicine and Biological Sciences, Chicago, IL 60637, USAjustinelee89@bsd.uchicago.edu (J.L.); spolster@uchicago.edu (S.P.P.);; 2 Center for Research Informatics, University of Chicago Medicine and Biological Sciences, Chicago, IL 60637, USA

**Keywords:** cerebral cavernous malformation, microRNAs, biomarkers, systematic review, hemorrhage, neurovascular diseases

## Abstract

Hemorrhagic neurovascular diseases, with high mortality and poor outcomes, urge novel biomarker discovery and therapeutic targets. Micro-ribonucleic acids (miRNAs) are potent post-transcriptional regulators of gene expression. They have been studied in association with disease states and implicated in mechanistic gene interactions in various pathologies. Their presence and stability in circulating fluids also suggest a role as biomarkers. This review summarizes the current state of knowledge about miRNAs in the context of cerebral cavernous malformations (CCMs), a disease involving cerebrovascular dysmorphism and hemorrhage, with known genetic underpinnings. We also review common and distinct miRNAs of CCM compared to other diseases with brain vascular dysmorphism and hemorrhage. A systematic search, following the Preferred Reporting Items for Systematic Reviews and Meta-Analyses guideline, queried all peer-reviewed articles published in English as of January 2025 and reported miRNAs associated with four hemorrhagic neurovascular diseases: CCM, arteriovenous malformations, moyamoya disease, and intracerebral hemorrhage. The PubMed systematic search retrieved 154 articles that met the inclusion criteria, reporting a total of 267 unique miRNAs identified in the literature on these four hemorrhagic neurovascular diseases. Of these 267 miRNAs, 164 were identified in preclinical studies, while 159 were identified in human subjects. Seventeen miRNAs were common to CCM and other hemorrhagic diseases. Common and unique disease-associated miRNAs in this systematic review motivate novel mechanistic hypotheses and have potential applications in diagnostic, predictive, prognostic, and therapeutic contexts of use. Much of current research can be considered hypothesis-generating, reflecting association rather than causation. Future areas of mechanistic investigation are proposed alongside approaches to analytic and clinical validations of contexts of use for biomarkers.

## 1. Introduction

Stroke is the second-leading cause of death worldwide, and neurological disorders are the leading cause of disability-adjusted life-years [1,2]. The global economic burden of stroke was estimated at USD 891 billion in 2017 and is predicted to increase to USD 2.31 trillion by 2050 [3]. Hemorrhagic stroke is associated with high mortality rates and worse outcomes than ischemic stroke [4]. Several vascular pathologies can cause brain bleeding, with varying degrees of understanding regarding their pathophysiologic mechanisms [5].

In 1993, Nobel laureates Ambros and Ruvkun first reported micro-ribonucleic acids (miRNAs) in post-transcriptional gene regulation [6,7]. Circulating miRNAs have since emerged as candidate biomarkers of clinical activity in cancer, and more recently in association with neurovascular disorders [8,9]. MiRNAs are small (19–25 bp), non-coding RNAs that regulate post-transcriptional gene expression via mRNA silencing [10]. Their direct relationship with the cellular transcriptome makes them key players in the regulation of intracellular signaling pathways and cell-to-cell communication [10]. Of interest, several studies indicate that miRNAs from pathological tissue are detectable in the blood flow or cerebrospinal fluid (CSF), suggesting their propensity to reflect tissue-specific clinical changes [11]. Furthermore, miRNAs have also been proven to be effective measures of treatment response [12]. MiRNAs can be leveraged as diagnostic and prognostic indicators of disease state and applied as monitoring biomarkers of drug effects; they have even been suggested as gene silencing therapies [8,13,14]. Several miRNA-based diagnostic tools are currently available to clinicians largely focused on cancer, but no miRNAs have been approved as therapies [15,16]. The role of miRNAs in neurovascular disease has only begun to be explored. Several miRNA discoveries have been reported in cerebral cavernous malformations (CCMs), a disease involving vascular dysmorphism and brain bleeding, where substantial progress has been made regarding its genetic underpinnings. Other neurovascular entities such as arteriovenous malformations (AVMs) and moyamoya disease (MMD) involve vascular dysmorphism primarily, with a lesser predisposition to bleeding, and allow the exploration of potentially common and distinct miRNAs. And of course, spontaneous intracerebral hemorrhage (ICH) offers an opportunity to identify miRNAs implicated in brain bleeding per se. We conduct a systematic review of miRNAs implicated in these pathologies. Commonalities may reveal new insights into the mechanisms of vascular dysmorphism and brain bleeding, which will pave the way toward identifying prime miRNA candidates for future study and clinical biomarker development. Distinct miRNAs may reflect unique and different mechanisms. We clarify knowledge gaps, identify cogent hypotheses based on this emerging knowledge, and pro-pose areas of future research.

## 2. Methods

**Figure 1:**
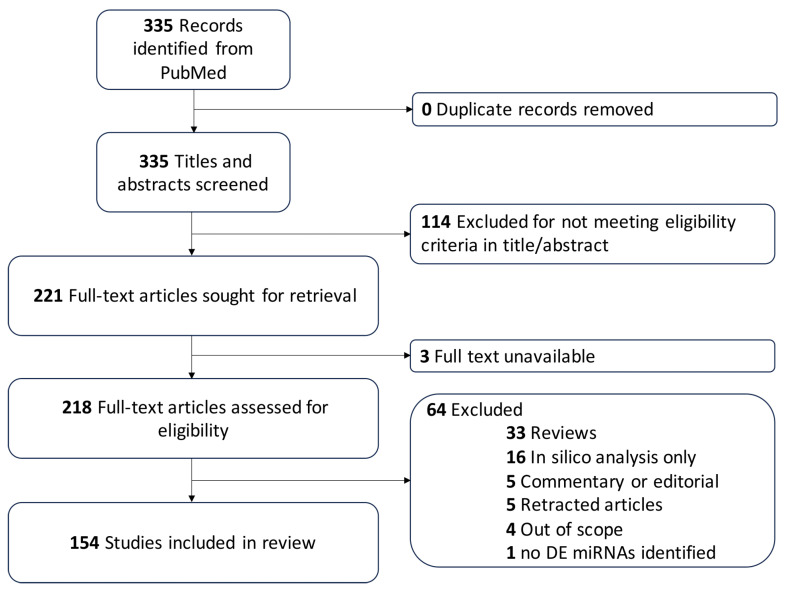
Preferred Reporting Items for Systematic Reviews and Meta-Analyses (PRISMA) flow diagram. Flow of information through the various phases of the systematic review. A comprehensive search on PubMed, following the PRISMA guidelines, queried 335 studies. No duplicate records were found. As they were not in English (*n* = 4) or not about miRNAs in CCM, AVM, MMD, or ICH (*n* = 110), 114 articles were excluded. In addition, 3 full texts were not retrievable. From the 218 full-text articles assessed for eligibility, 64 were excluded because they were either reviews (*n* = 33), in silico analyses (*n* = 16), commentary or editorial (*n* = 5), retracted articles (*n* = 5), or out of scope (*n* = 4). One article did not find any differentially expressed (DE) miRNAs in a mouse ICH model. Finally, 154 studies were included in this systematic review.

A comprehensive search through PubMed was conducted in January 2025 using the Preferred Reporting Items for Systematic Reviews and Meta-Analyses reporting guidelines (Figure 1) [17]. The search strategy is included in the Supplementary Material.

Clinical and preclinical studies written in English that reported miRNAs associated with CCMs, AVMs, MMD, or ICH were included. Reviews, commentaries, editorials, and studies solely focused on in silico-predicted miRNAs were excluded.

Eight researchers (A.Sr., A.J., C.B., J.K., R.J.A.-F., A.B., S.R., J.I.) first independently screened the abstracts and titles. Three team members (C.B., A.B., A.J.) performed data extraction independently from selected queried articles. Any disagreements between the reviewers were resolved by group consensus of at least three other authors (R.G., J.K., A.Sr., S.R., R.J.A.-F., J.I.).

Data extraction was conducted methodically using predefined criteria to ensure precision and consistency, capturing key elements such as miRNAs, the type of experimental model, whether target validation was mechanistic or predictive, the biological processes involved, control cohort in clinical studies, the sample type, and the directionality of miRNA expression. Data synthesis then followed a structured approach that utilized Venn diagrams, narrative synthesis, and thematic analysis to comprehensively integrate and interpret the findings across the included studies. The systematic review was not registered with a public registry.

Ingenuity Pathway Analysis (IPA) was further performed for differentially expressed (DE) miRNAs from various samples in preclinical and clinical studies common between those with CCM and AVM, MMD, or ICH (i.e., unsupervised analysis), limiting the query to only DE genes of lesional CCM tissue (i.e., supervised analysis) [13,18,19,20]. For more information, refer to the Supplementary Material.

## 3. Results

The PubMed search retrieved 335 manuscripts. After an initial screening of titles and abstracts, 221 articles met the inclusion criteria. Full-text analysis led to the exclusion of 67 studies, resulting in a final selection of 154 studies (Figure 1).

The studies included five preclinical [18,21,22,23,24] (Supplementary File S1) and three clinical studies [23,25,26] (Supplementary File S2) on CCM, three preclinical [27,28,29] (Supplementary File S3) and six clinical [28,29,30,31,32,33] (Supplementary File S4) studies on AVM, and four preclinical [34,35,36,37] (Supplementary File S5) and fourteen clinical studies [36,37,38,39,40,41,42,43,44,45,46,47,48,49] (Supplementary File S6) for MMD. Finally, 115 preclinical [14,50,51,52,53,54,55,56,57,58,59,60,61,62,63,64,65,66,67,68,69,70,71,72,73,74,75,76,77,78,79,80,81,82,83,84,85,86,87,88,89,90,91,92,93,94,95,96,97,98,99,100,101,102,103,104,105,106,107,108,109,110,111,112,113,114,115,116,117,118,119,120,121,122,123,124,125,126,127,128,129,130,131,132,133,134,135,136,137,138,139,140,141,142,143,144,145,146,147,148,149,150,151,152,153,154,155,156,157,158,159,160,161,162,163] (Supplementary File S7) and 26 clinical studies [14,148,149,150,151,152,153,154,155,156,157,158,159,160,161,162,163,164,165,166,167,168,169,170,171,172] (Supplementary File S8) on ICH were also included. Some studies included both preclinical and clinical components, contributing to the overall count.

The clinical studies reported a total of 1359 patients with CCM (*n* = 46; age = 29.91 years ± 18.49, range = [24–45]), AVM (*n* = 15; age = 27.53 years ± 9.82, range = [24–31]), MMD (*n* = 391; age = 34.18 years ± 16.48, range = [12–53]), or ICH (*n* = 907; age = 57.62 years ± 9.43, range = [47–68]) pathologies (Supplementary Files S2, S4, S6 and S8).

**Figure 2:**
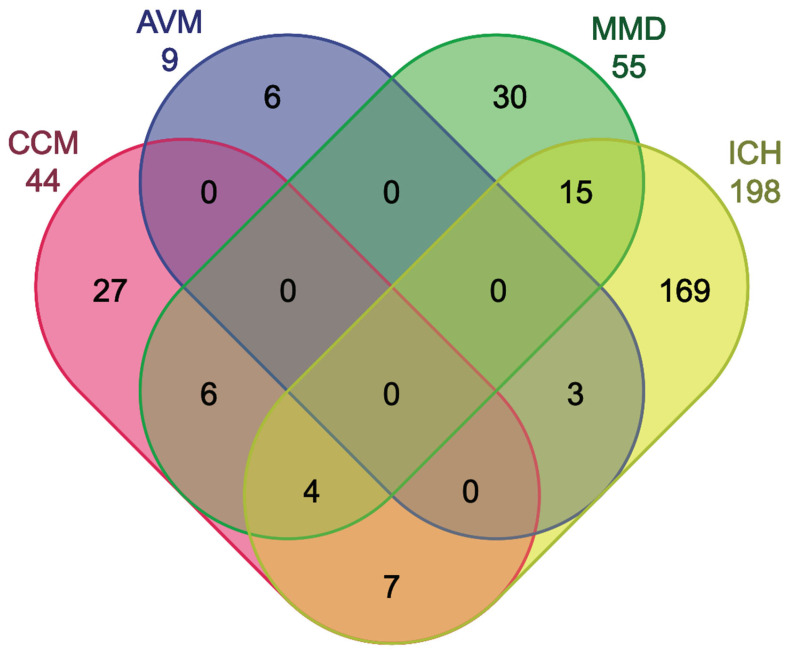
Unique and shared miRNAs across CCM, AVM, MMD, and ICH studies. Venn diagram illustrating the common and distinct miRNAs identified in the four studied pathologies.

The systematic search identified a total of 267 unique miRNAs, of which 44 were found in CCM, 9 in AVM, 55 in MMD, and 198 in ICH studies (Figure 2, Supplementary File S9). Of interest, 10 miRNAs identified in preclinical studies on CCM disease were also reported dysregulated in CCM patients. In addition, 3 miRNAs reported in preclinical studies on AVM, 3 in MMD, and 28 in ICH were also dysregulated in patients (Supplementary File S10). Using CCM as a paradigm, six miRNAs overlapped with MMD and seven with ICH, and four were common across CCM, MMD, and ICH (Figure 2). Further comparisons showed that 27 miRNAs identified either in a preclinical context or in patients were only observed in CCM disease (Supplementary File S9). No miRNAs were found to overlap between CCM and AVM (Figure 2).

### 3.1. Cerebral Cavernous Malformations

CCMs are vascular lesions characterized by clusters of leaky, immature vessels that predispose patients to a lifetime risk of hemorrhagic stroke, seizures, and focal neurologic deficits [173,174,175]. CCMs affect approximately 0.5% of the population and occur in either a sporadic or genetically inherited (familial) form [25,176]. CCM pathobiology includes loss of vascular endothelial cell (EC) junctions [177], neuroimmune cell activity [178], increased endothelial-to-mesenchymal stem cell transition [179], and aberrations in apoptosis, cytoskeletal organization, and cell proliferation processes [180,181].

#### 3.1.1. Dysregulated Intracellular miRNAs Are Mechanistically Tied to Vascular Pathobiology

Several studies have reported the mechanistic ties of miRNAs to CCM pathobiology and to clinical course using human plasma. Li et al. (2020) have reported in a cell line of mouse-derived ECs that the levels of *miR-27a* modulate the activity of VE-cadherin, a major endothelial adhesion molecule (Supplementary File S1) [22]. The inhibition of this binding using CD5-2 normalized the vasculature within CCMs [22]. Of interest, *miR-27a* has been previously identified as being upregulated in the brain tissue of CCM patients [25]. Upregulation of *miR-*27*a* results in loss of vascular integrity at the blood–brain barrier (BBB) [22]. In lesional tissue, upregulation of *miR-*27*a* may be related to altered redox homeostasis and oxidative stress conditions implicated in CCM pathogenesis (Supplementary File S1) [182,183,184,185,186,187]. A similar binding between *miR-425-5p* and the 3′ UTR of *CCM3* was identified in human ECs (Supplementary File S1) [21]. This binding was further tied to downstream inhibition of Notch signaling and activation of p38/VEGF signaling [21].

#### 3.1.2. CCM miRNAs Are Differentially Expressed in Mouse and Human Tissue

A preclinical study in murine models sought to identify circulating miRNAs reflecting the *Ccm3* genotype [18]. Koskimaki et al. (2019) showed lower plasma levels of *miR-3472a*, which targets *Cand2* (Supplementary File S1) [18]. Several other reports have queried CCM-relevant miRNAs using DE analysis in surgically resected human tissue [24,25]. Kar et al. (2017) identified five additional miRNAs as being downregulated in the brain tissue of CCM patients compared to healthy controls (Supplementary File S2) [25]. In a similar study, Schwefel et al. (2019) later investigated the DE of intracellular miRNAs in ECs resected from *CCM3* patients [24]. This study identified seven dysregulated miRNAs, with follow-up gene ontology analyses showing enriched pathways related to vascular development and aging (Supplementary File S1) [24]. Further analyses showed that three of these five miRNAs targeted genes such as *VEGF*, *MAPK1*, *RHOA*, *and ENG* [25].

While these intracellular miRNAs show putative association with CCM pathways and genotypes, they failed to appear as in vivo markers in analyses of CCM patient plasma [23].

#### 3.1.3. Circulating miRNAs as Clinical Markers of CCM and Symptomatic Hemorrhage

Several plasma miRNAs have been shown to be up- or downregulated when compared between CCM patients and healthy controls (Supplementary File S2) [13,23,25]. Analyses of the differential plasma miRNome identified nine homologous DE common miRNAs between mouse models of *Ccm1/3* and neurovascular units (NVUs) resected from patients with similar genotypes (Supplementary Files S1 and S2) [23]. The targets of these DE miRNAs included major CCM-associated pathways, including PI3K-Akt signaling, focal adhesion, HIF-1, cell adhesion molecules, and Rap1 signaling [23]. This reverse-translational finding not only suggested the ability of circulating miRNA to signal disease states but also generated viable targets for future investigations into preclinical models of CCM gene restoration therapy [23]. Additionally, this same study showed that circulating miRNAs were able to predict new lesion formation in CCM patients, further iterating the potential for plasma miRNAs to act as markers of disease progression [23].

Having established the biomarker viability of circulating miRNAs, the plasma miRNome of CCM patients has been integrated with additional circulating molecules to achieve higher specificity and selectivity models [26]. One such integrative study found that the ability of a diagnostic association model to distinguish patients who had sustained a symptomatic bleed from those who had not was improved by more than 20% after adding the plasma levels of DE miRNAs compared to a model with only plasma proteins [26]. Of interest, *miR-20a-5p*, *miR-25-3p*, and *miR-486-5p* showed mechanistic links to CCM pathways such as HIF-1, MAPK, PI3K-Akt, Rap1, and VEGF signaling (Supplementary File S2) [26]. Furthermore, recent evidence suggests that polymorphic variations in genetic modifiers (e.g., polymorphic cytochrome P450 enzymes) observed in CCM patients may be used for personalized medicine strategies and to improve hemorrhage risk stratification [188].

### 3.2. Arteriovenous Malformations

AVMs are abnormal connections between arteries and veins, predisposing patients to a lifetime risk of hemorrhagic stroke and seizures [175]. The mechanisms of AVM pathogenesis are poorly understood beyond vascular wall remodeling changes and feeding artery flow rates [28]. A stronger understanding of these mechanisms could improve clinical care, as current treatments rely on surgical resection or radiation therapy.

In in vitro assays of EC lines, *miR-18a* was found to protect against aberrant angiogenic processes by increasing thrombospondin-1 and decreasing VEGF (Supplementary File S3) [29,31,32]. In addition, increased activity of *miR-18a* was associated with decreased extracellular matrix disruption by decreasing matrix metalloproteinases activity, preventing vascular breakdown [29]. Of interest, several experiments have further shown that Argonaute-2 promotes the entry of this *miR-18a* into brain tissue (Supplementary File S4) [31]. Other studies have also shown that KRAS mutant ECs of AVMs increased exosomal *miR-3131* levels, which promoted endothelial–mesenchymal transition via PICK1 (Supplementary File S4) [33]. In addition, Chen et al. (2022) studied altered blood flow within AVMs using an arteriovenous high-blood-flow shunt rat model [27]. The results showed the upregulation of *miR-134-5p* and downregulation of *miR-204-3p* in the vascular wall remodeling process (Supplementary File S3) [27].

Several studies in patients have suggested the role of miRNAs in the pathophysiology and clinical course of AVM. Huang et al. (2017) showed decreased levels of *miR-137* and *miR-195* in the smooth muscle cells of human AVMs, which are important for cell survival and protecting the NVU from hemorrhage (Supplementary File S4) [28]. Finally, studies with the plasma of AVM patients have identified *miR-7-5p*, *miR-199a-5p*, and *miR-200b-3p* as central in VEGF signaling (Supplementary File S4) [30].

### 3.3. Moyamoya Disease

MMD is characterized by stenosis and occlusion of blood vessels within the circle of Willis, namely the intracranial internal carotid artery, and the middle and anterior cerebral arteries [189]. In response to occlusive arteriopathy, abnormal small vessel networks form near the base of the brain [189], which can cause ischemia and hemorrhage.

#### 3.3.1. MiRNAs Are Mechanistically Associated with MMD

Two miRNAs, *miR-125a-3p* and *let-7c*, have been shown to regulate the “synthetic” phenotype in vascular smooth muscle cells (VSMCs), which can lead to fibrocellular hyperplasia and intimal thickening [35,36]. Such fibrocellular hyperplasia and intimal thickening were accompanied by increased cell migration, proliferation, and extracellular matrix deposition [35,36]. In addition, Liu et al. (2022) showed in an in vitro ischemic MMD model that circZXDC sponges *miR-125a-3p,* increasing VSMC transition to the synthetic state (Supplementary File S5) [35]. Finally, this miRNA was also shown to regulate VSMC transdifferentiation by targeting *ABCC6*, a gene that induces ER stress and is highly expressed in MMD vessels (Supplementary File S5) [35]. Ma et al. (2023) showed that the levels of *let-7c* were elevated in the plasma of MMD patients when compared to controls [36]. This miRNA has also been upregulated in human ECs under hypoxic conditions (Supplementary File S5) [36]. In both in vitro and in vivo models, *let-7c* activation of TLR7 was shown to induce VSMC transition into the synthetic phenotype through Akt/mTOR signaling, ultimately leading to MMD-related vascular wall remodeling and intimal hyperplasia [36]. In addition, *let-7c* has been shown to target *RNF213*, a gene implicated in MMD pathogenesis [49]. Dysregulation of *RNF213* affects wall formation and vessel growth (Supplementary File S6) [49]. An *Rnf213* deficiency in mice led to thinner vessel walls after carotid artery ligation [49]. This result suggests that *RNF213* may be associated with angiogenesis [49]. Finally, *RNF213* has also been associated with MMD risk in human genome studies [49].

#### 3.3.2. Circulating miRNAs Are Differentially Expressed in MMD Patients

Of interest, *let-7c* was also found to be DE in both MMD patient plasma and serum when compared to controls (Supplementary File S6) [36,49]. Dai et al. (2014) also identified DE miRNAs in the serum of MMD patients, four of which were validated and found to have mechanistic implications in MMD pathogenesis [38].

Additional analysis of the circulating miRNAs identified by Dai et al. (2014) with DE lncRNA and mRNA data from another cohort of MMD patients revealed *miR-107* and *miR-423-5p* to be core regulators of vascular remodeling and cell proliferation under hypoxic conditions (Supplementary File S6) [38,39]. Several other studies identified DE miRNAs as potential MMD biomarkers (Supplementary File S6) [40,45,46]. Of interest, Uchino et al. (2018) reported that *miR-6722-3p* and *miR-328-3p* differentiated MMD from non-MMD cases in a study of MMD-discordant monozygotic twins (Supplementary File S6) [45]. Finally, Wang et al. (2021) developed a prognostic model with four miRNAs, upregulated in the CSF of MMD patients, which were able to predict neoangiogenic collateral vessel formation after indirect bypass surgery [46].

### 3.4. Intracerebral Hemorrhage

Non-traumatic ICH is the second most common type of stroke, representing 15% of cases and showing the highest mortality [190]. Primary ICH constitutes 85% of cases and typically results from the rupture of arteries and arterioles due to chronic hypertension or cerebral amyloid angiopathy [85,190,191]. Secondary ICH may arise from an underlying vascular malformation [192]. Evidence suggests that miRNAs modulate genes related to ICH pathological processes such as vascular integrity, oxidative stress, and neurodegeneration [85].

#### 3.4.1. MiRNAs Are Shown to Modulate Brain Vascular Integrity and Adhesion

In rat ICH models, *miR-18* and *miR-124* have been shown to affect bleeding and neurological outcomes by regulating the production of tight junction proteins (Supplementary File S7) [85,90]. Furthermore, *miR-24-1-5p* and *miR-126* have been shown to act as crucial regulators of HIF-1α and VEGFA in ECs within the PI3K/Akt signaling pathway (Supplementary File S7) [56,59]. Their dysregulation has been implicated in the breakdown of tight junction protein expression, cellular viability, and angiogenesis [56,59]. In in vitro and in vivo murine models of ICH, overexpression of *miR-6838-5p* and *miR-126* (Supplementary File S7) has also been shown to reduce apoptosis and neuroinflammation while enhancing tight junction expression [75,153]. This modulation improves BBB integrity by inhibiting VEGFA [153], which, if increased, leads to EC apoptosis and exacerbates ICH pathology [75].

Liu et al. (2022) recently reported in rat models that an in situ upregulation of *miR-126* following ICH decreased glial fibrillary acidic protein expression, neuroinflammation, and brain edema by downregulating ZEB1 (Supplementary File S7) [82]. Of interest, in ICH, *miR-126a-3p* promoted bone marrow mesenchymal stem cell differentiation into vascular ECs in vivo and in vitro (Supplementary File S7) [106]. This in turn produced a decrease in brain edema and BBB permeability via enhanced expression of tight junction proteins [106]. Precise therapeutic miRNA delivery may modulate ICH permeability across various pathways, cell types, and developmental stages [193].

#### 3.4.2. MiRNAs Are Shown to Modulate Apoptosis/Ferroptosis

Two in vitro studies have demonstrated that targeting acyl-CoA synthetase long-chain family member 4 using *miR-29a-3p* and *miR-106b-5p* reduced oxidative stress and ferroptosis (i.e., iron-dependent cell death) in hippocampal neurons and increased capillary EC survival (Supplementary File S7) [52,53]. Kong et al. (2021) showed that administering *antagomiR-23a-3p* in vivo reduced ferroptosis in rat ICH models by activating NRF2 signaling, which mitigated neuroinflammation (Supplementary File S7) [76]. In addition, oxidative stress, inflammation, and apoptosis have also been linked to *miR-93-5p* [158]. Upregulating NRF2, an important antioxidant response regulator, reduced apoptosis in vitro via transforming growth factor-β1, which acts as a competitive endogenous RNA of *miR-93-5p* (Supplementary File S7) [158]. In a rat ICH model, monomethyl fumarate pretreatment increased *miR-139* expression and led to upregulation of NRF2 and downregulation of NF-κB pathways (Supplementary File S7) [95].

Inhibition of the TRAF6/NF-κB axis by *miR-194-5p* and *miR-150-3p* has also been shown to reduce inflammasome activation and apoptosis in mouse ICH models (Supplementary File S7) [97,102]. Inhibiting NLRP3 inflammasomes using *miR-194-5p* and *miR-223* improved brain edema and neurological outcomes (Supplementary File S7) [102,131]. Additionally, inhibition of *let-7c* in the insulin-like growth factor receptor 1 pathway decreased cell death, neuroinflammation, and brain edema, ultimately improving neurological outcomes (Supplementary File S7) [74].

#### 3.4.3. MiRNAs Are Shown to Modulate Neuroinflammation After ICH in Microglia

An upregulation of *miR-7* and *miR-140-5p* mitigated secondary ICH inflammation through inhibition of the TLR4 pathway (Supplementary File S7) [110,143]. Secondary neuroinflammation and gliosis in perihematomal tissue are important mediators of neurological outcomes following ICH [194]. Microglial infiltration and neuroinflammation correlate with endoplasmic reticulum (ER) stress markers like HSPA5, which have been shown to be mitigated by overexpression of *miR-181b* (Supplementary File S7) [116]. In addition, an *miR-124* mimic has been reported to promote in vitro and in vivo microglia M2 polarization in perihematomal tissue, attenuating neuron apoptosis and neuroinflammation (Supplementary File S7) [133]. The importance of C/EBP-α in perihematomal tissue was further highlighted in an in vitro study with microglial cells isolated from ICH patients, showing that *miR-367* overexpression promoted microglia M2 polarization and decreased neuroinflammation (Supplementary File S7) [157]. Similarly, increased microglia M2 polarization has also been observed following *let-7a* overexpression through decreasing protein levels of CKIP-1 (Supplementary File S7) [130]. Upregulation of *miR-183-5p* and *miR-590-5p* decreased microglial-mediated inflammation and attenuated brain injury in ICH by inhibiting heme oxygenase and Pellino-1, respectively (Supplementary File S7) [65,112]. Additionally, the knockdown of lncRNA metastasis suppressor-1 upregulated *miR-709* and decreased secondary brain injury in both in vitro and in vivo mouse ICH models by decreasing microglial activation and proinflammatory cytokines (Supplementary File S7) [54].

In the lesional bed, blood degradation products cause microglia-mediated metabolic and oxidative stress in neurons through exosome transfer of *miR-383-3p* (Supplementary File S7) [118]. Of interest, hemoglobin-induced autophagy of microglia was attenuated with *miR-144* inhibitors in vivo by upregulating the mTOR pathway (Supplementary File S7) [117]. The Akt/mTOR pathway has been implicated in ICH as *miR-23b* upregulation increased both p-Akt and p-mTOR expression, resulting in negative regulation of inositol polyphosphate multikinase-mediated autophagy (Supplementary File S7) [69]. Paradoxically, Nie et al. (2020) showed that hemoglobin degradation products can decrease inflammatory signaling in microglia by downregulating *miR-331-3p* (Supplementary File S7) [86].

#### 3.4.4. MiRNAs Are Shown to Modulate Neuroinflammation After ICH in Neurons

Several studies have demonstrated that PTEN inhibition with upregulation of the PI3K signaling pathway has neurological benefits [61,81,145]. For instance, an overexpression of *miR-29a* promoted axonal regeneration and enhanced neurological outcomes in a rat ICH model by targeting *Pten* (Supplementary File S7) [145]. PTEN downregulation via L-lysine-induced overexpression of *miR-575* was also shown to be neuroprotective in mouse ICH models (Supplementary File S7) [55]. Liu et al. (2021) reported that an upregulation of the PI3K pathway with hypoxia-induced *miR-326* overexpression enhanced stem cell therapy in ICH by increasing autophagy and improving neuronal survival (Supplementary File S7) [81]. Conversely, downregulating the PI3K/AKT pathway increased neuroinflammation, neuronal apoptosis, BBB permeability, and microglial activation [61,136].

Neurodegeneration following ICH has been associated with multiple pathways and miRNAs [71,93,125]. In a rat model, *miR-146a* overexpression decreased neuroinflammation, brain edema, neuronal cell death, and oxidative stress, by modulating NF-κB signaling (Supplementary File S7) [71,125]. Early after ICH, intracellular levels of Ca^2+^ increase dramatically, causing ER stress and decreasing anti-apoptotic proteins [93]. Shen et al. (2021) reported that *miR-124* overexpression in a rodent ICH model reduced Ca^2+^ overload in neurons, mitigating neurodegeneration by targeting calmodulin-dependent protein kinase II (Supplementary File S7) [93]. Upregulating *Bcl-2* via *miR-133b* modified mesenchymal stromal cell-derived exosomes, reduced neuronal apoptosis by suppressing RHOA, and activated the ERK1/2/CREB pathway (Supplementary File S7) [94]. Similarly, sevoflurane decreased neuronal apoptosis in a mouse ICH model by enhancing *miR-133b* expression, which targets *FOXO4*, which increased BCL2 expression (Supplementary File S7) [78]. Anti-apoptotic pathway-targeting miRNA therapies could thus potentially be leveraged to prevent neurodegeneration in ICH.

#### 3.4.5. MiRNAs Are Shown to Modulate Neuroinflammation After ICH in Immune Cells

In a mouse ICH model, upregulation of *miR-125b-2-3p* decreased neuroinflammation by attenuating mast cell degranulation (Supplementary File S7) [129]. In addition, decreased expression of *miR-181a* in peripheral blood mononuclear cells (PBMCs) of a swine ICH model was shown to correlate with increased neuroinflammation via an interconnected network of monocytes and IL-8 (Supplementary File S7) [101]. Higher PBMC counts, particularly monocytes, are associated with increased 30-day fatality in ICH patients [195].

#### 3.4.6. Circulating miRNAs Are Dysregulated in ICH Patients

In ICH patients, various circulating miRNAs have been found dysregulated compared to control subjects (Supplementary File S8) [109,149,152,165,167,170,172]. Notably, *miR-124* serum levels correlate with neurological severity and functional outcomes (Supplementary File S7) [149]. Of interest, *miR-21-5p* has shown contradictory roles in studies reporting both upregulation and downregulation in cerebral hematoma samples as well as in peripheral blood and hematoma samples (Supplementary File S8) [87,170]. In a case–control study of 106 ICH cases, plasma levels of *miR-223*, *miR-155*, and *miR-145* were increased while *miR-181b* was decreased compared to healthy subjects (Supplementary File S8) [165]. Serum levels of *miR-23a-3p* and *miR-130a* have been found upregulated in ICH patients, while most DE miRNAs are downregulated in ICH patients (Supplementary File S8) [109,152]. Yang et al. (2021) suggest that ICH severity could be rather explained by single-nucleotide polymorphisms, as decreased serum and CSF levels of *miR-143* in patients with rs41291957 genotype were associated with poor neurological outcomes and increased proinflammatory factors (Supplementary File S8) [14]. Finally, Zheng et al. (2012) found that hematoma expansion or stability after ICH can be classified with 100% accuracy using 10 DE plasma miRNAs (Supplementary File S8) [171].

## 4. Discussion

### 4.1. MiRNA Commonalities of CCM and AVM

This systematic review did not identify any documented dysregulated miRNAs common to both CCMs and AVMs. Since these two neurovascular diseases have different genetic and molecular origins, the miRNA regulatory networks may therefore not overlap. In addition, these two vascular malformations have phenotypic differences [28,176]. CCMs typically represent low-flow lesions that can leak or bleed at low pressure [176]. On the contrary, AVMs are high-flow lesions characterized by direct arteriovenous shunting that may modulate different endothelial remodeling processes geared toward coping with excessive shear and hemodynamic stress [27]. Of interest, Lee et al. (2024) showed an upregulation of *miR-135b-5p*, under hypoxic conditions within the ECs, suggesting a role of this miRNA during the physiopathogenesis of AVMs [196]. In addition, there are a limited number of preclinical and human CCM and AVM studies reporting DE miRNAs. These studies show heterogeneity in inclusion criteria that introduce variability in miRNA findings and complicate cross-study comparisons. Finally, the documented studies have a small sample size that can result in underpowered analyses, making it difficult to detect subtle differences in miRNA expression.

### 4.2. MiRNA Commonalities Between CCM and MMD

**Figure 3:**
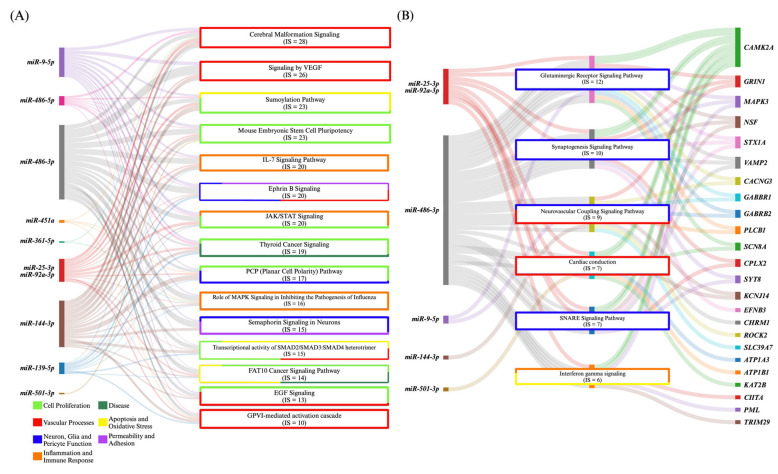
Ingenuity Pathway Analysis (IPA) of differentially expressed (DE) miRNAs common between cerebral cavernous malformation (CCM) and moyamoya disease. The IPA analyses of the gene targets and their associated pathways of *miR-9-5p*, *miR-486-5p*, *miR-486-3p*, *miR-451a*, *miR-361-5p*, *miR-25-3p*, *miR-92a-3p*, *miR-144-3p*, *miR-139-5p*, and *miR-501-3p* common between cerebral cavernous malformation (CCM) and moyamoya disease (**A**) identified 360 pathways (*p* < 0.01, false discovery rate [FDR] corrected) related to vascular, cell proliferation, and inflammation and immune response processes. Only pathways with an interaction score (IS) of 10 and a gene ratio of 0.265 are displayed. (**B**) Further analyses identified 201 enriched pathways (*p* < 0.01, FDR corrected) with gene targets (i.e., of the miRNAs mentioned above) that have been shown to be dysregulated in the transcriptome of neurovascular units of surgically resected CCMs. This result suggests common pathogenic processes between the CCM and moyamoya diseases. Only pathways with an IS of 6 and a gene ratio of 0.2 are displayed. IPA considered *miR-25-3p* and *miR-92a-3p* as the same entities as they harbor the same seed sequence.

This review identified a total of ten DE miRNAs in both CCM and MMD. Six of them, *miR-139-5p*, *miR-361-5p*, *miR-486-3p*, *miR-486-5p*, *miR-501-3p*, and *miR-92a-*3p, were only DE in CCM and MMD, while four (discussed separately) were also commonly DE between CCM, MMD, and ICH (Figure 3). Schwefel et al. (2019) demonstrated that *miR-139-5p* targets CXCR4, which has been shown to activate the PI3K/Akt, PLC, and ERK1/2 signaling pathways, all of which contribute to cell migration and proliferation [24,197]. Although *miR-139-5p* was upregulated in *CCM3*^-/-^ endothelium, its inhibition did not restore *CXCR4* expression or reverse endothelial dysmorphism [24].

While the majority of these miRNAs were upregulated in MMD [40,42,43,46], they were predominantly downregulated in CCM [25,26], suggesting fundamental differences in their underlying molecular mechanisms. Huang et al. (2023) observed that elevated plasma levels of 10 miRNAs, including *miR-501-3p*, had a high accuracy for diagnosing MMD [40]. This miRNA has been associated with actin cytoskeleton modulation via MAPK signaling, and increased levels have been shown to promote vascular sclerosis through tight junction protein-1 disruption [40,198]. Wang et al. (2021) showed that increased CSF levels of *miR-486-3p* and *miR-92a-3p* were able to predict angiogenesis in MMD patients with high accuracy [46]. In MMD, stenosis of large arteries causes collateral vessel formation through aberrant VEGF-mediated angiogenesis, induced by ischemia [199]. In CCM, increased VEGF similarly causes dysmorphic angiogenesis with high permeability [20]. However, decreased plasma levels of VEGF have been observed to predispose patients to cavernous angioma with symptomatic hemorrhage (CASH) or lesion growth [200]. Of interest, *miR-486-3p*, *miR-486-5p*, and *miR-92a-3p* together with *miR-501-3p* were found to be downregulated in the plasma of CASH patients [26]. Taken together, these results suggest that cytoskeletal, junctional, and angiogenic factors regulated by miRNAs may influence bleeding risk and serve as potential clinical biomarkers. Although mechanistic and predictive studies of *these miRNAs* are lacking in CCM and MMD research, common DE miRNAs identified in clinical studies between CCM and MMD may underscore a common pathological angiogenic process in both, inherent to vascular dysmorphism.

### 4.3. MiRNA Commonalities of CCM and ICH

**Figure 4:**
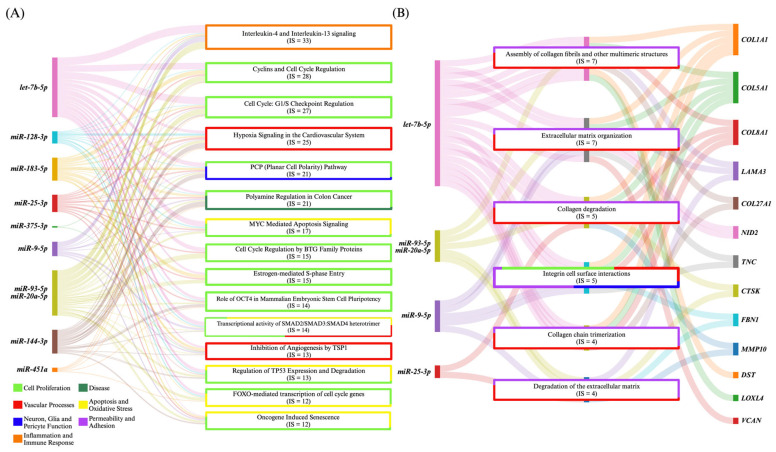
Ingenuity Pathway Analysis (IPA) of differentially expressed (DE) miRNAs common between cerebral cavernous malformation (CCM) and intracerebral hemorrhage. The IPA analyses of the gene targets and their associated pathways of *let-7b-5p*, *miR-128-3p*, *miR-183-5p*, *miR-25-3p*, *miR-375-3p*, *miR-9-5p*, *miR-93-5p*, *miR-20a-5p*, *miR-144-3p*, *miR-451a*, and *miR-27a*, commonly differently expressed between cerebral cavernous malformation (CCM) and intracerebral hemorrhage, (**A**) identified 450 pathways (*p* < 0.05, false discovery rate [FDR] corrected) related to cell proliferation and vascular processes. Only pathways showing an interaction score (IS) of 12 and a gene ratio of 0.29 are displayed. (**B**) Further analyses identified 190 enriched pathways (*p* < 0.01, FDR corrected) with gene targets (i.e., of the miRNAs mentioned above) that have been shown to be dysregulated in the transcriptome of neurovascular units of surgically resected CCMs. This result suggests common pathogenic processes between the CCM disease and intracerebral hemorrhage. Only pathways with an IS of 4 and a gene ratio of 0.29 are displayed. IPA considered *miR-93-5p* and *miR-20a-5p* as the same entities due to the same seed sequence, while *miR-27a* was unmapped.

CCM and ICH are both characterized by a failure of the NVU, with disruption of the vascular wall and blood extravasation occurring in small vessels [190,201]. In addition to the four common miRNAs in MMD, ICH, and CCM, this review identified *let-7b-5p*, *miR-128-3p*, *miR-183-5p*, *miR-20a-5p*, *miR-27a*, *miR-375-3p*, *and miR-93-5p* as commonly dysregulated in CCM and ICH, reflecting potential common molecular underpinnings and therapeutic targets for both diseases (Figure 4).

Vascular processes and permeability and adhesion pathways such as extracellular matrix organization and collagen degradation pathways emerged with IPA of ICH and CCM miRNAs and the CCM transcriptome (Figure 4). Although its exact role remains unclear, *let-7b-5p* targets *MLLT4* and may influence vascular integrity [25]. By contrast, *miR-128-3p* has shown therapeutic promise in ICH models, where its administration dampens microglial inflammatory response by repressing TXNIP expression [62]. Yet in CCM, *miR-128-3p* is paradoxically upregulated in a *Ccm1* mouse model and downregulated in the plasma of *CCM3* patients [23]. This duality highlights how the same miRNA can differentially regulate vascular stability and inflammation depending on lesion subtype or stage. Notably, *miR-128-3p* also targets *IGF1* and *NRXN1,* which have been linked to PI3K–Akt, HIF-1 signaling, and cell adhesion [23]. In addition, *miR-183-5p* has been shown to be downregulated in the brain tissue of ICH murine models as well as in the plasma of CCM CASH patients. Exogenous delivery of *miR-183-5p* reduced neuroinflammation, oxidative stress, and functional deficits in mouse ICH models via modulation of Nrf2 and NLRP3 pathways [26,58,112].

*MiR-20a-5p*, *miR-27a*, and *miR-93-5p* have been shown to modulate endothelial proliferation and vessel stability [22,162]. *MiR-20a-5p* and *miR-93-5p* have been found downregulated both in the plasma of CCM and blood of ICH patients [23,26,162], while *miR-27a* was upregulated in the plasma of ICH patients and in an in vivo CCM model [22,167]. In a mouse ICH model, *miR-20a-5p* overexpression attenuated hemorrhagic injury by regulating the HIF1α/VEGFA signaling pathway [162]. Meanwhile, *miR-27a* and *miR-93-5p* are downstream modulators of two important CCM transcription factors, KLF2 and KLF4 [22,167,202]. Alterations in these pathways have been shown to decrease intracellular levels of VE-cadherin and disrupt vascular integrity [22]. In fact, inhibition of the *miR-27a*/VE-cadherin interaction rescues CCM lesion development [22]. In addition, *miR-93-5p* targets VEGFA, ADAMTS5, ROCK2, and MAP3K14 and may affect both angiogenesis and lesion stability [23]. A downregulation of *miR-93-5p* has been shown in in vitro ICH models to decrease apoptosis via upregulation of NRF2, an important regulator of antioxidant response [158]. Of interest, KRIT1 loss of function is known to cause increased oxidative stress with responsive upregulation of NRF2 [203,204]. However, chronic upregulation of this antioxidant pathway predisposes CCM patients to additional oxidative insults via an increase in reactive oxygen species, as well as aberrant cell death [203,204]. These shared miRNAs highlight overlapping pathways of endothelial dysfunction, inflammation, and oxidative injury in ICH and cavernous malformations. Future investigations will clarify their mechanistic roles and therapeutic value in stabilizing the NVU across diverse cerebrovascular diseases.

### 4.4. MiRNA Commonalities of MMD, ICH, and CCM

This review also showed that *miR-9-5p*, *miR-144-3p*, *miR-25-3p*, and *miR-451a* were identified as commonly dysregulated across CCM, MMD, and ICH. Recent studies show that 13.5% of *miR-9-5p* gene targets appear in the human CCM lesional transcriptome and are tied to cell adhesion molecules and focal adhesion (including TNC, VAV3, and VCAN) [23]. Endothelial secretion of ADAMTS5, together with the cleavage of versican (i.e., encoded by *Vcan*), has been identified as a downstream mechanism in CCM pathogenesis [205]. In addition, increased ADAMTS5 expression in endothelial cells appears to act with CCM1 loss of function, resulting in larger vascular malformations [23].

For instance, *miR-144-3p* and *miR-25-3p* have been linked to apoptotic and oxidative stress pathways, processes central to hemorrhagic injury [61,79]. In a rat ICH model, *miR-144-3p* overexpression aggravated brain edema and neurobehavioral disorders by targeting *Fpr2*, associated with the PI3K/AKT pathway [61]. In a mouse ICH model, lower levels of *miR-25-3p* induced upregulation of NOX4 and the production of hydrogen peroxide and ER stress [79]. The increased expression observed in ICH models might reflect a compensatory or pathogenic response to acute hemorrhage and oxidative damage.

A consistent, albeit opposite, expression pattern in MMD/CCM versus ICH models points to a shared molecular framework with disease-specific contexts that modulate miRNA activity and downstream vascular responses. Overall, data suggests that targeting these miRNAs may hold therapeutic promise, but clinical translation requires a nuanced understanding of when and how each miRNA exerts its functions. Further studies are needed to validate these regulatory roles in larger patient cohorts with more comparable control groups, elucidate cell-type-specific mechanisms, and explore the potential for miRNA-based interventions to improve outcomes.

### 4.5. Distinct miRNAs in CCM, AVM, MMD, and ICH and Their Implications

Preclinical mouse models and clinical plasma samples suggest that *miR-20b-5p*, *miR-323-3p*, *miR-369-5p*, *miR-410-3p*, and *miR-487b-3p* were only upregulated in CCM disease [23]. These miRNAs appear to converge on pathways critical for vascular homeostasis and inflammation, including Rap1 and NF-κB signaling [23,206,207]. For example, *miR-20b-5p* targets *VEGFA* and *ADAMTS5*, impacting Rap1 signaling, which is integral to EC migration, proliferation, and membrane localization of *CCM1/KRIT1* [23]. *MiR-323-3p and miR-410-3p* have been linked to elevated EC apoptosis or inflammatory cascades in vascular diseases, underscoring their broader involvement in pathological vascular remodeling [206,207]. Taken together, these findings suggest that the upregulated miRNAs in CCM may serve both as biomarkers of disease progression and as potential targets for therapeutic intervention.

Clinical and preclinical findings reported that *miR-137* and miR-195* are downregulated in AVM tissue [28]. In vivo mouse models further show that mimics of these miRNAs suppress aberrant VSMC migration and tube formation [28]. Notably, *miR-137* and miR-195* modulate key signaling pathways such as including VEGF, PI3K/Akt, and MAPK/ERK that are essential for normal vascular development [28]. Therapeutic strategies aimed at restoring *miR-137* and miR-195* may help promote proper vasculogenesis, inhibit aberrant vascular growth, and ultimately protect against the occurrence or progression of AVMs [28].

Among the miRNAs uniquely associated with MMD, *miR-125a-3p* and *miR-6760-5p* each show consistent differential expression across at least two independent studies [35,37,38,47]. *MiR-125a-3p* is downregulated in both in vitro and clinical samples, and mechanistic data suggest that this decrease leads to ABCC6 overexpression, which correlates with intimal thickening and ER stress [35,38]. In contrast, *miR-6760-5p*—which antagonizes the angiogenic activity of YAP1 through the Hippo signaling pathway—is upregulated in both preclinical and clinical MMD samples, where it reduces cell proliferation, movement, and tube formation [37,47]. Notably, *miR-6760-5p* also exhibits strong diagnostic potential, with an area under the curve of 0.918 in distinguishing MMD patients from healthy controls [47]. Together, these findings highlight *miR-125a-3p* and *miR-6760-5p* as critical molecular players in MMD pathogenesis and potential biomarkers or therapeutic targets.

In addition, *miR-124*, *miR-124-3p*, *miR-155*, *miR-181b*, and *miR-195-5p* were reported in both preclinical and clinical ICH studies, appearing in at least three distinct investigations [63,64,83,85,91,93,99,100,107,116,122,123,133,142,149,150,154,159,165,166,169]. Consistent with clinical observations, *miR-124* circulating plasma level appears to exhibit a biphasic pattern [159]. In acute ICH murine models, an upregulation of *miR-124* suppresses AGO2 [159] and C/EBP-α and fosters an M2-dominant microglial phenotype that lessens inflammatory damage [133]. Conversely, in later phases, the downregulation of *miR-124* beneficially increases ferroportin levels, thereby reducing iron overload and related injury [149]. Although *miR-124* and *miR-124-3p* represent different strand maturation stages, the 3p strand has been reported to target distinct genes, including *TRAF6* and *MTF1* [107,150]. Overexpression of *miR-124-3p* has been shown to attenuate oxidative stress as well as proinflammatory responses in microglia and astrocytes [107,150,154]. Notably, clinical data indicate that serum levels of *miR-124* rise sharply after ICH onset, followed by a decline as recovery ensues—an expression trajectory that may reflect ongoing tissue repair mechanisms [159].

Beyond the *miR-124* family, additional miRNAs consistently display impactful roles in ICH outcomes. *MiR-155* is predominantly upregulated across multiple models, potentiating inflammatory mediators such as IL-1β, IL-6, and TNF-α, whereas inhibiting this pathway reduces oxidative stress and improves neurological function [63,123,142,165]. Conversely, *miR-181b* and *miR-195-5p* exhibit more protective profiles [83,99,100,122,169]. An increase in *miR-181b* levels counteracts inflammation and edema [122,169]. Similarly, *miR-195-5p* upregulation mitigates apoptosis, dampens oxidative stress, and decreases MMP-2/9 activity to preserve the BBB [83,99,100]. Collectively, these findings underscore the therapeutic potential of miRNA modulation for regulating iron metabolism, neuronal survival, inflammatory cascades, and vascular integrity in ICH.

## 5. Limitations

Several limitations must be acknowledged while interpreting the results. Most of the papers do not consider different disease phenotypes, genetic modifiers, and environmental or therapeutic factors. The majority of the studies are retrospective, with suboptimal controls, and subject to selection and interpretation biases. Secondly, animal models do not always accurately mimic human conditions. However, homologous miRNAs have been shown in preclinical models of CCM and patients [23]. Furthermore, the difference in tissue sampling and the comparison of their miRNome can lead to the identification of miRNAs that may not be shared across all three diseases. Finally, many of the associations do not prove causality, nor do they implicate specific mechanisms of miRNAs in disease pathogenesis.

## 6. Conclusions and Future Directions

MiRNAs have risen to the forefront of neurovascular biomarker research and hold the potential to become powerful tools in diagnostic and prognostic evaluations. Common miRNAs may reflect shared pathogenic mechanisms between hemorrhagic neurovascular disorders occurring during their natural history. Different vascular dysmorphisms predisposing patients to brain bleeding reflect unique and common molecular aberrations, and these are reflected in the associated miRNAs. Brain bleeding proper, regardless of vascular pathology, involves molecular cascades that reflect miRNA interactions and associations.

Much of the research herein can be considered hypothesis-generating and compels future mechanistic studies of individual miRNAs in tissue and fluids, and in relation to disease gene aberrations. These studies will clarify the biologic plausibility of miRNA associations and identify the potential roles of miRNAs as gene silencing therapies.

Biomarker associations require analytic validations to confirm molecular sensitivity and specificity related to miRNA levels and not mere differential expression. Research should address the stability of these molecules, their potential association with sex, age, and co-morbidities, and their change in different disease states. Finally, clinical validations of biomarker contexts of use require well-designed prospective studies with rigorous controls.

## Supplementary Materials

The following supporting information can be downloaded at: https://www.mdpi.com/article/10.3390/ijms26083794/s1.
