# Supplementary material for: A Systematic Review of MicroRNAs in Hemorrhagic Neurovascular Disease: Cerebral Cavernous Malformations as a Paradigm
Source: Int J Mol Sci. 2025 Apr 17;26(8):3794. doi: 10.3390/ijms26083794 (PMC12028044; doi:10.3390/ijms26083794)
Supplement: Supplementary file 1 [file ijms-26-03794-s001.zip › supplementary_material_030425.pdf]

Supplementary Material

A Systematic Review of MicroRNAs in Hemorrhagic Neurovascular Disease: Cerebral Cavernous Malformations as a Paradigm

Running Title: MicroRNAs in Hemorrhagic Neurovascular Disease

Roberto J. Alcazar-Felix[1], Aditya Jhaveri[1], Javed Iqbal[1], Abhinav Srinath[1], Carolyn Bennett[1], Akash Bindal[1], Diana Vera Cruz[2], Sharbel Romanos[1], Stephanie Hage[1], Agnieszka Stadnik[1], Justine Lee[1], Rhonda Lightle[1], Robert Shenkar[1], Janne Koskimäki[1], Sean Polster[1], Romuald Girard[†1], Issam A. Awad[†1]

† **Equal contribution:** Romuald Girard and Issam A. Awad share senior authorship.

[1]Neurovascular Surgery Program, Department of Neurological Surgery, University of Chicago Medicine and Biological Sciences, Chicago, IL, USA

[2]Center for Research Informatics, University of Chicago Medicine and Biological Sciences, Chicago, IL, USA

Supplemental Methods

Queried PubMed terms

The following concepts were predefined using the PubMed Advanced Search Builder query box:

Concept #1: "cerebr*"[All Fields] OR "brain"[MeSH Terms] OR "brain*"[All Fields].

Concept #2: "hemangioma, cavernous"[MeSH Terms] OR "cavernous angioma*"[All Fields] OR "cavernous malformation*"[All Fields] OR "cavernous hemangioma*"[All Fields] OR "cavernoma*"[All Fields].

Concept #3: arteriovenous malformation OR AVM.

Concept #4: intracerebral hemorrhage OR ICH.

Concept #5: moyamoya disease.

Concept #6: "MicroRNAs"[MeSH Terms] OR "microrna*"[All Fields] OR "mirna*"[All Fields] OR "micro rna*"[All Fields] OR "small rna*"[All Fields] OR "mi-rna*"[All Fields] OR "micro-rna*"[All Fields].

Using the prespecified concepts, the following query was searched: ((#1 AND #2) OR (#1 AND #3) OR #4 OR #5) AND #6.

Unsupervised and Supervised Ingenuity Pathway Analysis (IPA)

Differentially expressed (DE) miRNAs of interest were isolated from various samples (e.g., plasma, serum, whole blood, cerebral spinal fluid, circulating leukocytes, extracellular vesicles [EV], resected tissue) between patients and control subjects in clinical studies, in pre-clinical study disease models and control animals, or between treated and non-treated cells. Gene targets with experimental evidence or high inference confidence for each DE miRNA of interest were identified using IPA (QIAGEN, Hilden, Germany) [1]. Unsupervised pathway analysis was first completed on all gene targets, assuming significance for all, using the IPA core platform (QIAGEN).

A supervised pathway analysis was then performed using the lesional human cerebral cavernous malformation (CCM) transcriptome [2-4], limiting the query to only the differently expressed genes (DEGs) ($p<0.05$, false discovery rate [FDR] corrected). The raw sequencing data is freely available in the National Center for Biotechnology Information Gene Expression Omnibus (GEO) database and is accessible through GEO series accession numbers GSE130176, GSE134005, and GSE134007.

An interaction score, defined as the number of unique miRNA–gene target pairs associated with a pathway, was calculated for both IPA analyses and each miRNA.

Supplemental Results

Unsupervised IPA identifies DEG targets of miRNAs within pathways

The unsupervised IPA identified 985 gene targets for 11 DE miRNAs common between cerebral cavernous malformation (CCM) and intracerebral hemorrhage (ICH). In addition, 1107 gene targets were found for the ten miRNAs common between CCM and moyamoya disease (MMD). Further IPA analyses identified 450 pathways for ICH and 360 for MMD ($p<0.01$, FDR corrected).

Supervised IPA identifies DEG targets of miRNAs within pathways enriched in CCM

The supervised pathway analyses focused on pathways involving only the gene targets of the common miRNAs that were also enriched within the transcriptome of lesional CCM

neurovascular units, consisting of 1,542 DEGs ($p<0.05$, FDR corrected) [2,3]. The 11 miRNAs common between CCM and ICH had 108 DEG targets. Furthermore, the 10 DE miRNAs common between CCM and MMD had 135 DEG targets. Additional analyses showed that the common miRNAs targeted CCM transcriptome genes in 190 pathways for ICH, and 201 for MMD ($p<0.01$, FDR corrected).

Supplemental References

1. Kramer, A.; Green, J.; Pollard, J., Jr.; Tugendreich, S. Causal analysis approaches in Ingenuity Pathway Analysis. *Bioinformatics* **2014**, *30*, 523-530, doi:10.1093/bioinformatics/btt703.
2. Lyne, S.B.; Girard, R.; Koskimaki, J.; Zeineddine, H.A.; Zhang, D.; Cao, Y.; Li, Y.; Stadnik, A.; Moore, T.; Lightle, R.; et al. Biomarkers of cavernous angioma with symptomatic hemorrhage. *JCI Insight* **2019**, *4*, doi:10.1172/jci.insight.128577.
3. Koskimaki, J.; Zhang, D.; Li, Y.; Saadat, L.; Moore, T.; Lightle, R.; Polster, S.P.; Carrion-Penagos, J.; Lyne, S.B.; Zeineddine, H.A.; et al. Transcriptome clarifies mechanisms of lesion genesis versus progression in models of Ccm3 cerebral cavernous malformations. *Acta Neuropathol Commun* **2019**, *7*, 132, doi:10.1186/s40478-019-0789-0.
4. Li, Y.; Girard, R.; Srinath, A.; Cruz, D.V.; Ciszewski, C.; Chen, C.; Lightle, R.; Romanos, S.; Sone, J.Y.; Moore, T.; et al. Transcriptomic signatures of individual cell types in cerebral cavernous malformation. *Cell Commun Signal* **2024**, *22*, 23, doi:10.1186/s12964-023-01301-2.
